# Using decision methods to examine the potential impact of intersectoral action programs

**DOI:** 10.1186/s13104-018-3609-x

**Published:** 2018-07-27

**Authors:** Wanrudee Isaranuwatchai, Ahmed M. Bayoumi, Emilie Renahy, Rebecca Cheff, Patricia O’Campo

**Affiliations:** 1 Centre for Excellence in Economic Analysis Research (CLEAR), The HUB, Li Ka Shing Knowledge Institute, St. Michael’s Hospital, 30 Bond Street, Toronto, ON M5B 1W8 Canada; 2 Institute of Health Policy, Management and Evaluation, University of Toronto, 155 College St, Toronto, ON M5T 3M6 Canada; 3 Centre for Urban Health Solutions, Li Ka Shing Knowledge Institute, St. Michael’s Hospital, 30 Bond Street, Toronto, ON M5B 1W8 Canada; 4 Department of Medicine, University of Toronto, 27 King’s College Cir, Toronto, ON M5S 1A1 Canada; 5 Division of General Internal Medicine, St. Michael’s Hospital, 30 Bond Street, Toronto, ON M5B 1W8 Canada; 6 Lea Roback Research Centre on Social Inequalities and Health, 1301, rue Sherbrooke Est, Montreal, QC H2L 1M3 Canada; 7 Département de médecine sociale et préventive, Université de Montréal, 2900 Edouard Montpetit Blvd, Montreal, QC H3T 1J4 Canada; 8 Wellesley Institute, 10 Alcorn Ave, Toronto, ON M4V 3B1 Canada; 9 Dalla Lana School of Public Health, University of Toronto, 27 King’s College Cir, Toronto, ON M5S 1A1 Canada

**Keywords:** Decision methods, Intersectoral action programs, Public health research, Cross-sectoral research, Economic evaluation, Portfolio analysis, Multiple-criteria decision analysis, Programme budgeting and marginal analysis

## Abstract

**Objectives:**

In public health today, there is a widespread call for intersectoral action (ISA) programs, in which two or more sectors cooperate to address a problem. This trend raises a question of how to appropriately assess the effectiveness and cost-effectiveness of ISA programs. To assess the impact of ISA, evaluation methods should provide a framework for simultaneously considering the impact of two or more interventions when selecting from a portfolio of programs. There is a gap in literature on such methods. In this research note, from a narrative review, we report and describe methods that could be useful for evaluating ISA programs. Subsequently, we present a hypothetical case study to demonstrate the use of these methods.

**Results:**

We identified four methods that have potential to assess the joint impact of multiple interventions: economic evaluation, portfolio analysis, multiple-criteria decision analysis, and programme budgeting and marginal analysis. To keep pace with the desire to use strong evidence to inform the selection and design of ISA programs, methods must evolve to support these initiatives. This research note seeks to begin a dialogue on existing decision methods which may be used to assist decision makers with funding and resource allocation decisions of ISA programs.

**Electronic supplementary material:**

The online version of this article (10.1186/s13104-018-3609-x) contains supplementary material, which is available to authorized users.

## Introduction

Two complementary ideas are manifest within public health evaluation today: using evidence to inform programs and thinking about intersectoral action (ISA). *First*, there is movement toward making programs and policies more evidence-based [1–3]. In contrast to clinical interventions, the use of evidence to inform the development and implementation of public health programs has been more gradual, in part because of their complexity [4, 5]. *Second*, there is a widespread call for ISA, in which two or more sectors cooperate to address a problem [6–8]. For example, the transportation and public health sectors might work together to increase urban cycling by increasing cycling routes for commuting and implementing programs to reduce unnecessary automobile idling [9, 10].

These two trends raise questions about how to assess the effectiveness and cost-effectiveness of ISA programs. One challenge is that such programs often have disparate health and non-health impacts without an obvious common metric for measuring outcomes. A second challenge is that existing methods often focus on the impact of a single program while decision-makers often need to choose among programs. Evaluation methods should provide a framework for simultaneously considering the impact of two or more interventions, especially when there are disparate outcomes both within and between sectors. For example, while a program may have multiple interventions to improve transportation policies (e.g., reducing traffic flow in favor of wider sidewalks) or promote food security, the wide range of outcomes within and across such programs poses challenges for evaluators when trying to assess the programs’ joint impact.

Several methods may facilitate the evaluation of multiple interventions with disparate outcomes [11–16]. These methods are generally not used to their full potential by governments in which each sector operates in its silo, focusing only on one aspect of the evaluation at a time.

We conducted a narrative review of methods to evaluate intra- and inter-sectoral interventions (see Additional file 1 for search strategy). We report the selected methods that we thought were most useful for evaluating ISA programs with disparate outcomes, including their strengths, limitations, and gaps that should be addressed by further research. Subsequently, we present a hypothetical case study to demonstrate the use of these methods. The identified methods could be applicable to interventions within one sector as well; however, the focus of this paper will be on ISA programs.

## Main text

### Available methods

We agree with a literature review that approaches to measure or estimate the social value of a single intervention and commented that there is “no silver bullet” [17]. The following paragraphs provide a high-level overview of *four* methods that have potential to assess the joint impact of multiple interventions: economic evaluation, portfolio analysis, multiple-criteria decision analysis (MCDA), and programme budgeting and marginal analysis (PBMA).

*Economic evaluations* within health care are primarily used to assess the efficiency of an intervention. *Cost*-*utility analysis* measures outcomes using a quality-adjusted life year (QALY), which integrates quality of life and length of life into a single measure [11]. The limitation lies in the need to summarize quality of life in a single number from conceptual and measurement aspects [11]. The application of QALY outside of the health sector is challenging as there are no well-established frameworks for measuring QALY.

*Cost*–*benefit analysis* assigns monetary values to both costs and outcomes. Such approach might make evaluation of non-health interventions easier because one can assign dollar values to both health outcomes (e.g., having healthy babies), and non-health outcomes (e.g., improving air pollution). However, they have limitations, e.g., some attributes may be difficult to value in dollar values and people’s stated willingness-to-pay may differ from their ability to pay (raising equity concern) [11, 18].

A hybrid method uses a *net benefit approach*, where interventions are valued using a cost-effectiveness framework with outcomes specific to the intervention [19]. The cost-effectiveness ratio is then “converted” into a net benefit measure using the societal willingness-to-pay [20].

*Portfolio analyses* address the question of how to combine interventions within or across programs to maximize a given objective [15, 16]. Such analyses start by defining an “objective function”, which may include only one outcome (e.g., the number of human immunodeficiency virus (HIV) infections prevented), multiple outcomes with similar measures (e.g., the number of life years saved from preventing HIV and hepatitis C), or multiple outcomes with distinct measures (e.g., the number of life years gained and the number of arrests averted). With more than one outcome, an explicit weight can be assigned to each outcome so that they can be combined into a single number. The analysis then used mathematical programming to determine the optimal allocation across sectors according to the objective functions, subject to any recognized constraints. Each set of objectives and constraints might define a different perspective. For example, maximizing QALYs focuses on health outcomes only, and maximizing outcomes that combine QALYs and non-health benefits reflects preferences for both health and non-health outcomes. The main limitation of the portfolio analyses comes from the challenge of defining an objective function and the information needs for complex constraints.

One method for defining the relative importance of outcomes is multiple-criteria decision analysis. MCDA facilitates the ranking or prioritizing of a set of interventions using criteria that have been identified as relevant by stakeholders [12]. This approach involves a series of steps from defining the problem and structuring the selected criteria to performing the analyses and dealing with uncertainty [13]. Decision-makers are engaged at each step. Many approaches have been used to score individual criteria and aggregate scores; some will yield objective functions that can be used for portfolio analyses. Examples of MCDA include prioritizing policies concerned with cardiovascular disease prevention and control [21], conducting a risk assessment to identify pollution sites [22], and identifying hospital sites that increases access [23]. While MCDA can compare interventions to each other, it cannot account for unspecified combinations of interventions. Moreover, MCDA is conducted upon a number of assumptions, e.g., the perspective of the analysis (e.g., public payer versus society) and the weights assigned to each criterion. Some outcomes (e.g., equity concerns) may be excluded if they were out of scope for the chosen perspective.

*Programme budgeting and marginal analysis* aims to maximize defined objectives when choosing from a range of interventions within a budget constraint [14, 24]. The key elements of PBMA are how to maximize the benefit through marginal analysis, an examination of the additional benefits of an activity compared to the additional costs incurred by that same activity, and how to minimize the opportunity cost (the benefit foregone by not selecting an intervention). The main benefit of PBMA is that it explicitly takes into account opportunity cost [24] and it allows for consideration of other health system objectives such as equity [25]. Limitations of PBMA include limited availability of data for each criterion and the difference between “expected benefit” versus “real benefits”, i.e., the potential benefits compared to the actual benefits. PBMA has been used in public health decision making [26–28]. For example, health authorities in the UK who were involved in purchasing decisions used PBMA to guide purchasing decision in health care [28].

### Hypothetical case study

Our hypothetical ISA programs are increasing bike lanes and decreasing homelessness through a Housing First intervention. For simplicity, we consider only one intervention in each program. Creating new bike lanes will have an impact on health, by improving physical fitness, and on the environment, by reducing air pollution. Bike lanes have mixed effects on transportation, decreasing commuting time for cyclists but increasing time for motorists. Housing First intervention improves health among homeless people by providing stable housing. To illustrate how we might jointly evaluate these two interventions, we have identified six features which differentiate the evaluation approaches (Table 1).

**Table 1:** Ability of proposed approaches to consider elements in the comparison of ISA programs

Elements to consider when comparing two ISA programs	EE	PA	MCDA	PBMA
Can the method explicitly define all outcomes in any 1 or 2 unit (e.g., benefits and costs)?	+	+	+	+
Can the method explicitly define more than two outcomes?	−	+	+	+
Can the findings be generalizable to other context?	+	±	±	−
Can the method consider budget constraints?	−	+	+	+
Can the method consider other program constraints?	−	+	+	+
Does the method include a deliberative process with relevant stakeholders?	−	−	+	+

*EE* economic evaluation, *PA* portfolio analysis, *MCDA* multiple-criteria decision analysis, *PBMA* programme budgeting and marginal analysis, *+* yes, *−* no, *±* occasionally

To conduct an economic evaluation (cost–benefit analysis), we value all outcomes in monetary units. In addition to intervention costs (for building bike lanes and housing units), we anticipate cost savings from preventing adverse health events. Other outcomes for the bike lane intervention are valued in monetary units, e.g., increased traffic time for people who drive. We can conduct a survey, using contingent valuation methods [18], to determine how much money people feel is required to compensate them for this increased traffic time. The Housing First intervention is valued by summing intervention costs, potential cost savings (e.g., from averted hospitalizations), and other outcomes (e.g., improved mental health). The net cost (costs minus benefits) of each intervention is then calculated and the intervention with the lowest cost is deemed favorable. Economic evaluation can be generalizable across settings. Nevertheless, economic evaluation does not usually consider budget constraints or other concerns that are context-specific (e.g., equity). Thus, economic evaluation is best viewed as an input into a deliberative decision making process rather than a method for structuring the deliberation.

Portfolio analysis quantifies costs and outcomes but they need not be in the same units. We define an objective function to compare the two interventions and constraints. For instance, the objective function for the Housing First intervention might maximize both the number of days housed and the health status for people. The objective function for the bike lane intervention might maximize the amount of time spent cycling and minimize motorists’ commuting time. We might impose financial constraints (e.g., maximum budget for each intervention), and non-monetary constraints (e.g., ensuring that each neighbourhood gets some service). The objective function may also specify the importance of outcomes, e.g., avoiding an hour spent in commuting may be viewed as half as important as gaining an hour of cycling time. To obtain such tradeoffs, we could survey the public using discrete choice experiments [29]. Mathematical programing is then used to determine the allocation of resources that meets the objectives. Portfolio analysis is a quantitative input into the deliberative process, although it considers a wider array of concerns. This analysis can be generalizable to other settings, but it does not address how such inputs are actually used to make decisions.

For MCDA, we first identify evaluation criteria for each intervention. In our example, we will include: *reach* (the size of the population each intervention will affect), *quality of life*, *health service utilization*, *stable housing*, *traffic*, *revenue*, and *pollution*. Next, we score each intervention against each criterion, assign a weight to each criterion, and finally produce a summary score for each intervention. We will engage relevant stakeholders to ensure that relevant criteria are being captured, the data are appropriate, and the outputs of the analyses yield helpful information. The intervention with the highest summary score would represent the optimal intervention. MCDA results depend on which criteria are selected, e.g., the bike lane intervention will receive a better score for reducing pollution than the Housing First intervention. Budget and other constraints can be included if these are chosen as evaluation criteria but are not always considered. Because the criteria and process can be specific to a decisional context, MCDA can more readily be part of a deliberative process.

In PBMA, we define decision criteria using stakeholder input while ensuring that we take into account budget/resource constraints, all within a formal decision review process. For our case study, we will define the same criteria as in MCDA. The two interventions are evaluated against each criterion and assigned a rank. Next, each intervention will be assessed relative to its cost and the budget constraint. Decision-makers are tasked with explicitly considering, within their budget, both what is gained at the margin (i.e., the incremental losses and gains) from funding an intervention and what is foregone by not funding an alternative intervention (e.g., how many fewer housing units will be constructed by building one additional bike lane). Decision-makers thus determine the optimal allocation across interventions and confirm feasibility. PBMA is a method that addresses multiple concerns and guides the deliberative process. However, it is more specific to local contexts by incorporating budget analyse. Consequently, PBMA findings are likely to be the least generalizable across settings.

## Limitations

This study describes methods that could be useful for evaluating ISA programs, and presents a hypothetical case study to demonstrate their use. Our narrative review identified four methods; however, future research could initiate emerging methods, building on these identified methods. To keep pace with the desire to use strong evidence to inform the selection and design of ISA programs, methods must evolve to support these initiatives. This research note seeks to begin a dialogue on existing decision methods which may, with some modifications, be used to assist decision-makers with funding and resource allocation decisions of intersectoral action programs.

## Additional file

**Additional file 1.** Search strategy for this study.

## Abbreviations

EE: economic evaluation
HIV: human immunodeficiency virus
ISA: intersectoral action
MCDA: multiple-criteria decision analysis
PA: portfolio analysis
PBMA: programme budgeting and marginal analysis
QALY: quality-adjusted life year
