# Supplementary material for: Using decision methods to examine the potential impact of intersectoral action programs
Source: BMC Res Notes. 2018 Jul 27;11:506. doi: 10.1186/s13104-018-3609-x (PMC6062875; doi:10.1186/s13104-018-3609-x)
Supplement: Supplementary file 1 — Additional file 1. Search strategy for this study. [file 13104_2018_3609_MOESM1_ESM.doc]

# ADDITIONAL FILE: SEARCH STRATEGY

We performed electronic searches in Web of Science Core Collection. We combined groups of keywords in different ways: (multiple, intersectoral, combined, coordinated, integrated) AND (intervention, polic*, program, action, evaluation, “policy intervention”, “social intervention”). The same strategy and keywords were applied to retrieve information from the grey literature using Google Scholar. We also conducted a forward citation search of the studies which cited the included articles [1, 2]. We limited our search to publication written in French or English, and published in 2000 onwards. To be eligible for inclusion, a document had to consider at least two interventions (including any program, policy, project or action) and use quantitative data and methodologies. Any academic or research field, any publication type (e.g., theoretical, primary and secondary data studies, reviews), any intervention design (e.g., randomized control trial or not; targeted or universal interventions) and any outcome was considered for inclusion.
